# Management of Hypertension in Patients Undergoing Peritoneal Dialysis: A Narrative Review

**DOI:** 10.7759/cureus.105215

**Published:** 2026-03-14

**Authors:** Ariv A Ramlal, Anum Naimat, Abhishek Ramakrishnan, Mazin Pattathil, Tarab Shahbaz, Yumna Tariq, Mounica Bodige, Zakia Irum, Vinay Dontul, Anisa Ali, Mohammed Jaber

**Affiliations:** 1 School of Medicine, University of the West Indies, St. Augustine, TTO; 2 Internal Medicine, Fatima Memorial Hospital (FMH) College of Medicine &amp; Dentistry, Lahore, PAK; 3 School of Medicine, University of Debrecen, Debrecen, HUN; 4 School of Medicine, Government Medical College Idukki, Idukki, IND; 5 School of Medicine, Lahore Medical and Dental College, Lahore, PAK; 6 School of Medicine, Dera Ghazi Khan Medical College, Dera Ghazi Khan, PAK; 7 School of Medicine, Malla Reddy Medical College for Women, Hyderabad, IND; 8 School of Medicine, Fatima Jinnah Medical University, Lahore, PAK; 9 Internal Medicine, Apollo Institute of Medical Sciences and Research, Hyderabad, IND; 10 Medicine, Royal Liverpool University Hospital, Liverpool, GBR

**Keywords:** ace inhibitors, ambulatory blood pressure monitoring, dialysis, hypertension, icodextrin, peritoneal dialysis, renin-angiotensin-aldosterone system, volume overload

## Abstract

Hypertension is the most common complication in patients with end-stage renal disease (ESRD) on dialysis and a major contributor to cardiovascular mortality. This narrative review explores the multifactorial pathophysiology of hypertension in peritoneal dialysis (PD) patients, including extracellular fluid overload, sodium and water imbalance, sympathetic overactivity, activation of the renin-angiotensin-aldosterone system (RAAS), loss of residual kidney function (RKF), and inflammation with endothelial dysfunction.

Current clinical perspectives on blood pressure (BP) management are reviewed, highlighting variations in BP targets, the U-shaped relationship between BP and mortality, and the need for individualized, volume-focused treatment goals. Diagnostic and monitoring strategies such as ambulatory BP monitoring (ABPM), home BP monitoring, standardized office measurements, bioimpedance spectroscopy, and lung ultrasound are discussed for assessing hypertension and volume status.

Management approaches include non-pharmacologic strategies, optimization of dry weight, dietary sodium restriction, dwell time adjustments, low-sodium dialysate, icodextrin use for improved ultrafiltration, and physical activity and pharmacologic therapies including ACE inhibitors, angiotensin receptor blockers (ARBs), calcium channel blockers, beta-blockers, diuretics, mineralocorticoid receptor antagonists (MRAs), and agents for resistant hypertension such as clonidine and moxonidine.

Special considerations, such as peritonitis, which can alter peritoneal membrane function and ultrafiltration, are also addressed. Emerging therapies, including sodium-glucose co-transporter 2 (SGLT2) inhibitors, novel PD solutions with reduced glucose content, digital BP monitoring platforms, and lung ultrasound for real-time fluid assessment, highlight evolving strategies in this field.

In conclusion, hypertension management in PD requires a personalized, volume-targeted, and multidisciplinary approach integrating optimized monitoring, pharmacologic and non-pharmacologic interventions, and supportive lifestyle measures to improve patient outcomes.

## Introduction and background

Hypertension is highly prevalent among patients with end-stage kidney disease (ESKD) receiving peritoneal dialysis (PD) and represents a major contributor to cardiovascular morbidity and mortality [1-3]. Despite its clinical significance, optimal blood pressure (BP) management in PD patients remains insufficiently defined because randomized controlled trials specifically addressing this group are limited, and treatment is complicated by variability in extracellular volume status and residual kidney function (RKF) [4]. Managing BP in this group is complicated by unique physiological factors, specifically the variability in extracellular volume status and residual kidney function (RKF) [4]. Unlike the rapid fluid shifts seen in hemodialysis, PD involves continuous but often incomplete sodium and water removal, making hypertension in these patients uniquely volume-dependent [2,4].

Accurate assessment of BP in dialysis patients presents a clinical challenge. Fluid shifts frequently influence clinic-based BP readings and may not reflect the true interdialytic BP burden. Evidence demonstrates that out-of-office BP measurements, including ambulatory blood pressure monitoring (ABPM) and home blood pressure (HBP) monitoring, correlate more strongly with target-organ damage, cardiovascular events, and mortality than clinic measurements [4-6].

Consequently, contemporary nephrology guidelines recommend incorporating home or ambulatory BP monitoring into routine clinical evaluation whenever feasible [4, 6].

Defining BP targets in patients with PD remains controversial. Observational studies demonstrate a U-shaped association between BP and mortality, where both extremes are associated with adverse outcomes [2, 4, 7]. However, lower clinic-based BP often reflects underlying cardiac dysfunction, malnutrition, or inflammation. Studies using out-of-office measurements show a more linear relationship between elevated BP and cardiovascular risk, supporting physiologic BP assessment when determining treatment goals [4-6]. Current recommendations generally suggest maintaining BP below 140/90 mmHg, while emphasizing individualized targets based on cardiovascular tolerance [4, 6].

In PD patients, hypertension is predominantly volume-driven by sodium retention and peritoneal membrane transport characteristics [4, 7]. Therefore, management should begin with non-pharmacologic strategies aimed at achieving euvolemia, including dietary sodium restriction, optimization of dialysis prescription, use of osmotic agents such as icodextrin, and preservation of RKF [3, 7]. Persistent fluid overload is associated with left ventricular hypertrophy and increased mortality even when pharmacologic BP control appears achieved [1-3].

Pharmacologic therapy is required in many patients after volume optimization. RAAS inhibitors are commonly recommended as first-line agents due to potential benefits in BP reduction, cardiovascular protection, and preservation of residual kidney function [8]. Additional medications, including calcium channel blockers and beta-blockers, are frequently required, although drug selection must consider electrolyte balance, peritoneal protein losses, and risk of hypotension [4].

Given the complex interplay between fluid status, cardiovascular disease, and RKF, hypertension management in PD requires an individualized physiology-based approach rather than the strict application of general population algorithms. Further prospective studies are needed to establish evidence-based BP targets and therapeutic strategies tailored specifically to the PD population. This review aims to synthesize current evidence on these diagnostic and therapeutic strategies, providing a physiology-based framework for managing hypertension specifically within the PD population.

## Review

Methods

This study was conducted to synthesize current evidence on hypertension management in peritoneal dialysis (PD) patients. A comprehensive literature search was performed across PubMed, MEDLINE, and Google Scholar for articles published between January 2000 and December 2024 (Table 1). The search strategy employed combinations of MeSH terms and keywords, including "peritoneal dialysis," "hypertension," "blood pressure monitoring," "volume overload," "residual kidney function," and "antihypertensive therapy."

**Table 1:** Literature search and selection flow PD: Peritoneal dialysis

Search Stage	Description	Number of Records
Identification	Records identified through PubMed, MEDLINE, and Google Scholar (2000–2024)	482
	Additional records identified through reference lists and clinical guidelines	35
Screening	Total records after removing duplicates	312
	Records excluded after title and abstract screening (not relevant to PD/Hypertension)	164
Eligibility	Full-text articles assessed for eligibility	148
	Full-text articles excluded (Focus on Hemodialysis only, non-English, or pilot data)	22
Included	Total studies included in the narrative synthesis	126

The selection process involved an initial screening of titles and abstracts, followed by a full-text review of 126 identified papers. Additionally, we manually reviewed the reference lists of key studies and consulted major clinical guidelines from the International Society for Peritoneal Dialysis (ISPD), KDIGO, and KDOQI. Studies were evaluated based on their clinical relevance to the pathophysiology, diagnosis, and therapeutic management of hypertension, specifically within the PD population.

Pathophysiology of hypertension in PD patients

Hypertension in patients undergoing peritoneal dialysis is multifactorial and largely driven by disturbances in volume regulation, neurohormonal activation, and vascular dysfunction. Major contributors include extracellular fluid overload, sodium and water imbalance, activation of the renin-angiotensin-aldosterone system (RAAS), sympathetic nervous system overactivity, loss of residual kidney function, and inflammation-related endothelial dysfunction.

The Role of Extracellular Fluid Overload

Extracellular fluid overload is a main contributing factor to hypertension in patients undergoing peritoneal dialysis [9]. Historically, foundational studies in a European cohort (n=1,092) identified that fluid overload is evident in 53.4% of PD patients [10]. Hypertension can result from excessive sodium and fluid intake, declining residual renal function, and suboptimal peritoneal ultrafiltration [11]. Increased salt intake pulls water into extracellular spaces, while long-term exposure to glucose-degradation products (GDPs) in dialysis solutions impairs the peritoneal membrane’s ultrafiltration capacity. Declining RKF reduces the kidney’s ability to excrete sodium and water, while peritoneal membrane dysfunction caused by long-term exposure to dialysis solutions may impair ultrafiltration capacity [12, 13]. These changes promote volume expansion, increase cardiac preload, and, in turn, elevate blood pressure.

Sodium and Water Imbalance

Sodium retention contributes significantly to extracellular fluid expansion and hypertension in PD patients. In chronic kidney disease (CKD), impaired renal excretion of sodium and water leads to progressive volume overload and elevated blood pressure [14].

Upregulation of the Renin-Angiotensin-Aldosterone System (RAAS)

In PD patients, reduced renal perfusion activates RAAS. While this mechanism is well-characterized in the broader CKD population, it is particularly relevant in PD due to the unique osmotic environment. Renin catalyzes the conversion of angiotensinogen into angiotensin I, which is subsequently transformed into angiotensin II by angiotensin-converting enzyme. Angiotensin II promotes vasoconstriction and triggers aldosterone secretion, resulting in retention of sodium and fluid. In CKD and PD patients, impaired renal feedback mechanisms lead to persistent RAAS activation despite elevated blood pressure [15]. It was found in a foundational study that renin levels in CKD patients are usually twice the normal amount [16]. This contributes to a greater RAAS effect, continuing the increase in blood pressure.

Sympathetic Overactivity

CKD and PD patients often experience sympathetic nervous system (SNS) overactivity. Angiotensin II enhances norepinephrine release and stimulates central sympathetic pathways like the rostral ventrolateral medulla (RVLM). Uremic toxins such as adenosine and urea can stimulate renal afferent nerves, increasing SNS outflow. Furthermore, reduced activity of renalase, an enzyme that breaks down catecholamines, leads to a surplus of circulating norepinephrine, further raising blood pressure [17]. Since increased blood pressure is a sympathetic response, this will lead to hypertension in PD patients.

Loss of Residual Kidney Function (RKF)

RKF supports solute clearance, sodium balance, and cardiovascular regulation [18]. Its loss in PD patients results in greater uremic toxin accumulation, poor sodium handling, and activation of both RAAS and SNS, which can lead to hypertension [17]. Studies show that patients with RKF loss have increased left ventricular mass and worse blood pressure control [12,18]. Phosphate retention due to reduced RKF may also cause vascular calcification, increasing arterial stiffness and promoting hypertension [12].

Inflammation and Endothelial Dysfunction

Chronic inflammation in PD patients can arise from endotoxemia, catheter biofilms, high glucose PD fluids, and adipose cytokines. Endotoxins stimulate Toll-like receptor 4 (TLR-4), which subsequently promotes inflammatory cytokine production, including IL-6 and TNF-α [19]. Inflammatory cytokines (IL-6, TNF-α) shift the vascular balance toward vasoconstriction via endothelin-1 [19-21]. It also activates RAAS and SNS, both of which increase blood pressure [19]. Oxidative stress and glucose degradation products impair NO synthesis, contributing further to endothelial dysfunction and hypertension.

Understanding these mechanisms is essential for developing effective diagnostic strategies and therapeutic approaches for hypertension in PD patients.

Blood pressure targets in PD patients

Review of Guidelines

Hypertension management in patients undergoing peritoneal dialysis is guided by recommendations from several nephrology organizations, including the International Society for Peritoneal Dialysis (ISPD), Kidney Disease: Improving Global Outcomes (KDIGO), and Kidney Disease Outcomes Quality Initiative (KDOQI). Although specific recommendations vary, these guidelines consistently emphasize careful blood pressure monitoring, optimization of volume status, and individualized treatment strategies to reduce cardiovascular risk in this population.

ISPD Guidelines (International Society for Peritoneal Dialysis): Hypertension affects more than 80% of patients undergoing peritoneal dialysis and is associated with adverse clinical outcomes [22, 23]. The guidelines advise regular blood pressure monitoring using home measurements at least weekly, in addition to assessment during clinic visits [24]. For PD patients with consistently elevated blood pressure (>140/90 mmHg), treatment is advised to maintain blood pressure below 140/90 mmHg [24]. Elevated systolic blood pressure has been associated with a higher risk of mortality among peritoneal dialysis (PD) populations (Hazard Ratio [HR] 1.18; 95% CI 1.02-1.36; p=0.02) [25]. However, findings from at least one observational study reported that systolic blood pressure levels of ≤110 mmHg were linked to increased mortality, whereas systolic values >120 mmHg demonstrated a protective effect [26]. Before initiating or increasing the dose of antihypertensive medications, it is recommended that volume status be optimized in hypertensive patients on PD [24]. This is because hypertension in PD patients is associated with volume overload [27].

KDIGO Guidelines (Kidney Disease: Improving Global Outcomes): The 2021 KDIGO Clinical Practice Guideline for Blood Pressure Management in Chronic Kidney Disease emphasizes the use of standardized office blood pressure measurements rather than routine office measurements for managing high blood pressure in adults [28]. Blood pressure measurements obtained outside the clinical setting, such as ambulatory blood pressure monitoring (ABPM) or home blood pressure monitoring (HBPM), may be used in combination with standardized office readings for the management of hypertension [28]. KDIGO recommends a systolic blood pressure target below 120 mmHg based on standardized office measurements (Table 2) [29].

**Table 2:** Key guideline recommendations for BP targets and measurement in peritoneal dialysis (PD) SBP: Systolic blood pressure; PD: Peritoneal dialysis

Guideline Body	Target BP (Systolic/Diastolic)	Recommended Measurement Method	Specific Considerations for PD Patients
Mann et al., 2021 [28]	Not applicable for dialysis patients	Standardized Office BP Measurement	The Guideline explicitly excludes recommendations for patients receiving dialysis.
Rabi et al., 2020 [29]	<140/90 mmHg	Weekly Home BP Monitoring (HBPM) and at each clinic visit	Acknowledges "U-shaped" association with mortality; SBP ≤110 mmHg associated with increased mortality, SBP >120 mmHg appeared protective.

KDOQI perspective: From the perspective of the Kidney Disease Outcomes Quality Initiative (KDOQI), accurate blood pressure assessment is emphasized across different settings, including office-based measurements, home monitoring, and ambulatory blood pressure monitoring. Regardless of the measurement environment, the use of validated devices and appropriately sized cuffs is essential to ensure reliable readings. Proper patient preparation and positioning are also necessary in both clinical and home settings to obtain accurate measurements. In addition, patients should be educated about correct blood pressure measurement techniques and encouraged to adhere to established guideline recommendations [30]. The KDOQI commentary further underscores the relevance of the 2017 American College of Cardiology/American Heart Association (ACC/AHA) guidelines in the management of blood pressure among individuals with chronic kidney disease [31].

Controversies and Lack of Uniform Target

There are some controversies regarding the management of blood pressure and treatment plans in patients undergoing peritoneal dialysis. Below are a few studies on the use of ACEIs and ARBs for preserving residual renal function. An RCT including 60 incident PD patients found that the use of angiotensin-converting enzyme inhibitors could slow the decline in residual renal function (RRF) [32]. Another study showed that patients undergoing PD with angiotensin receptor blockers could preserve RRF, compared with the control groups [33]. However, a recent retrospective study conducted in the United States suggested that the use of ACE inhibitors or angiotensin receptor blockers may not significantly slow the decline of residual renal function in patients undergoing peritoneal dialysis [34]. Similarly, an observational cohort study of individuals initiating peritoneal dialysis in the Netherlands reported no significant renoprotective benefit associated with ACE inhibitor or angiotensin receptor blocker therapy [35].

Importance of Individualized Targets

Volume status: In patients receiving peritoneal dialysis, particularly those who are anuric, fluid overload may occur when the removal of sodium and water is inadequate relative to dietary intake [36-38]. Fluid overload has been associated with hypertension and left ventricular hypertrophy (LVH). Conversely, excessive fluid removal may lead to intradialytic hypotension and tissue ischemia, which can result in cardiac stunning, potential loss of cerebral white matter, and deterioration of residual renal function (RRF) [39-41]. Therefore, routine evaluation of fluid status is essential in clinical practice. Bioimpedance spectroscopy (BIS) represents a practical tool for assessing dry weight in dialysis patients. One study evaluating incident PD patients (n=1,092) reported that 56.4% were fluid overloaded at a cutoff value of 1.1 L, whereas 38.7% were normovolemic and 4.9% were volume depleted (p<0.001) [41]. Additionally, a large international cohort study monitoring fluid status in peritoneal dialysis patients over several years observed a modest but statistically significant improvement following initiation of peritoneal dialysis [42].

Comorbidities

Glycemic control in patients with diabetes on PD: According to the 2010 US Renal Data System, the overall one- and two-year survival rates for patients on PD were 83% and 67.2%, respectively, and a slightly lower rate of 80.3% and 61.7% in the diabetes subgroup [43]. A Canadian study reported one- and two-year survival rates of 94% and 89% among patients without diabetes, compared with 91% and 76% in those with diabetes [44]. Evidence from individual centers in Asia and Europe similarly demonstrated significantly lower survival rates in peritoneal dialysis patients with diabetes compared to those without diabetes [45]. In a study including 2,798 patients, a time-averaged HbA1c level ≥8% was identified as being associated with the highest risk of all-cause mortality [46]. Another investigation showed that glycemic control had greater clinical relevance in patients younger than 60 years [47]. Infection was identified as a major cause of mortality among individuals with HbA1c ≥8% [48]. These findings indicate that poor glycemic control in peritoneal dialysis patients adversely affects survival, partly due to an increased susceptibility to infection.

A study on non-diabetic patients, who began continuous ambulatory peritoneal dialysis, measured fasting blood sugar levels four weeks after starting treatment, showed 23.4% of patients had elevated fasting blood sugars [49].

Heart failure in PD patients: Cardiovascular disease represents the primary cause of mortality in individuals with end-stage renal disease (ESRD). It encompasses conditions such as acute myocardial infarction, cerebrovascular events, heart failure, persistent arrhythmias, and sudden cardiac death, with heart failure being among the most frequently observed complications. The presence of heart failure at the time of dialysis initiation is a strong predictor of both short-term (90-day) and long-term mortality [50, 51]. Additionally, a prior history of heart failure has been shown to increase the risk of cardiovascular death in patients undergoing peritoneal dialysis by more than threefold [52].

Major predictors of heart failure in patients receiving long-term peritoneal dialysis include hypertension, new or recurrent heart failure, and fluid overload. Reduced sodium and fluid removal have been associated with poorer survival outcomes in peritoneal dialysis patients, and those with hypertension are at greater risk of hospitalization due to cardiovascular events [53].

Metabolic syndrome in PD patients: In the last few decades, the incidence of metabolic syndrome in PD patients has been on the rise. Various studies suggest there is substantial variation between countries and ethnicities, but an overall impression is that the prevalence is higher than that in the general population. Prevention of metabolic syndrome in PD patients can be achieved through exercise, dietary modification, and by reducing glucose load [54].

Diagnostic and monitoring strategies

Office BP vs. Home BP vs. Ambulatory BP

Several studies have evaluated the optimal methods for measuring blood pressure in PD patients. They proved that both clinic and home blood pressure readings were similarly effective at identifying high blood pressure when compared to ambulatory readings. Specifically, one-week averaged home systolic BP was just as accurate as standardized clinic measurements in diagnosing hypertension. While both methods tended to slightly overestimate ambulatory BP, home monitoring offers a reliable alternative for assessing blood pressure in these patients [5].

Routine home blood pressure measurements obtained by patients undergoing peritoneal dialysis offer the benefit of a potentially more effective approach to blood pressure monitoring in hypertension management [55]. Comparative studies evaluating the diagnostic performance of various BP monitoring methods are presented in Table 2.

There are studies suggesting that routine home blood pressure readings are less accurate than standardized clinical measurements in reflecting daytime ambulatory blood pressure (Table 3) [23, 56, 57]; this discrepancy may be due to the absence of standardized protocols for home BP monitoring. Ambulatory BP monitoring is considered the gold standard method for managing hypertension in PD patients [55]. The Italian Cooperative Peritoneal Dialysis Study reported a non-dipping blood pressure pattern in 53% of the 504 participants assessed using this method [22]. Because ambulatory BP monitoring may not always be readily available, office-based BP measurement is recommended as the standard approach. Additionally, the use of validated home blood pressure devices is encouraged to enhance the reliability of home measurements [55].

**Table 3:** Studies investigating the effectiveness of various BP monitoring techniques for diagnostic evaluation in individuals receiving peritoneal dialysis. LV, left ventricular; 95% CI, 95% confidence interval A BpTRU records BP automatically without an observer.

Author	Year	N	Test Method	Reference Method	Study Results
Wang et al. [57]	2001	31	Clinic BP 10-d morning home BP	Ambulatory BP monitoring	The association of clinic systolic BP with daytime systolic BP (r=0.81; p<0.05) was stronger than the association of home systolic BP with daytime systolic BP (r=0.62; p<0.05)
Koc et al. [23]	2002	74	Clinic BP	Ambulatory BP monitoring	Twenty-four–hour ambulatory blood pressure monitoring identified left ventricular hypertrophy, whereas clinic blood pressure measurements showed no correlation with markers of target organ damage.
O’Shaughnessy et al. [56]	2013	17	Clinic blood pressure measurements, BpTRU-generated blood pressure readings, and seven-day morning home blood pressure monitoring.	Ambulatory BP monitoring	Home systolic blood pressure measurements were found to overestimate daytime systolic values by 14.2 mmHg (95% CI: 4.3–21.4 mmHg), while routine clinic BpTRU-derived readings provided a closer estimate of daytime blood pressure.

Assessment of Volume Status

Excess fluid in peritoneal dialysis patients can lead to serious complications like high blood pressure, stiff arteries, and an enlarged heart, all of which increase the risk of death, particularly from heart and brain-related events. Currently, there's no universally agreed-upon definition for ideal body weight in PD patients; it's likely the weight at which the body's extracellular fluid volume is normal. While various methods exist to gauge hydration, many have limitations. Direct measurements of body water are accurate but costly and time-consuming. Although levels of natriuretic peptides (like BNP) can indicate hydration changes, they're also influenced by heart issues and can build up in kidney failure. Bioimpedance emerges as a promising tool; it's accurate, consistent, affordable, and non-invasive, offering a good assessment of hydration. Ultimately, a combination of clinical evaluation, careful tracking of body weight, urine output, sodium and fluid intake, bioimpedance monitoring, and natriuretic peptide levels can help keep PD patients adequately hydrated [58].

Lung ultrasound is another technique to measure volume overload in the critical lung area. B-lines from lung US correlate with echocardiographic parameters but not with bioelectrical impedance analysis (BIA) measurements. As BIA and lung US estimate fluid overload in different compartments, they can be complementary tools for volume status assessment [59].

Role of Residual Urine Output

In a cross-sectional study of 195 patients undergoing continuous ambulatory peritoneal dialysis, participants were categorised as anuric, oliguric, and urine output (UO) > 400 ml. It showed that extracellular water among the three groups was not statistically significant, which suggested that residual renal function may not be as important as expected in maintaining good volume status. This finding indicates that reducing sodium and fluid consumption may play a more significant role in achieving improved blood pressure control in these patients [60].

In a retrospective cohort study of 202 patients, 58 with anuria and 144 patients without anuria, adjusted for age, follow-up periods, presence of diabetes, ultrafiltration volumes, albumin, haemoglobin, the peritonitis rate, and technique survival were not different between the groups (p > 0.05). But patient survival rates were better in the anuric group (p < 0.05) [61].

Sodium and Hypertension in Peritoneal Dialysis Patients

A prospective, randomized, double-blind clinical trial demonstrated that average daily sodium removal at 12 weeks was 1.18 g greater with low-sodium peritoneal dialysis solution compared with standard-sodium solution (p < 0.001). Blood pressure showed minimal change with the standard-sodium solution but declined with the low-sodium formulation, resulting in reduced use of antihypertensive medications. The noninferiority of total Kt/Vurea of low-sodium PD solution (2.53+/-0.89) over standard-sodium PD solution(2.97+/-1.58), however, could not be proved [62]. Nevertheless, more research is needed to determine the safest and most effective formulations and uses of these solutions to prevent complications like hyponatremia (low blood sodium) or excessively low blood pressure [63].

Role of Echocardiography and Cardiovascular Risk Assessment

A cross-sectional study evaluating abdominal aortic calcification (AAC) scores for predicting coronary artery disease (CAD) in middle-aged patients undergoing peritoneal dialysis (n=187) found that AAC scores were significantly higher in individuals with CAD (9.7±7.6, n=41) compared with those without CAD (5.5±6.1, n=146) (p<0.001). Increasing age (p=0.002), diabetes (p=0.002), hypertension (p=0.032), and lower serum potassium levels (p=0.012) were significantly associated with elevated AAC scores and increased CAD risk [64].

A prospective cohort study examining the relationship between left ventricular fractional shortening (LVFS) and cardiovascular events (CVE) showed that patients in tertile 3 had the lowest incidence of CVEs (24.5% vs 31.6% vs 43%, p<0.05). After adjustment for confounding variables in this PD-specific cohort, the tertile 3 group demonstrated a 45.1% reduction in cardiovascular event (CVE) risk compared with the tertile 1 group (Sub-distribution Hazard Ratio [SHR] 0.549; 95% CI 0.395-0.762; p<0.001) [65].

Accurate assessment of blood pressure and volume status forms the foundation for appropriate therapeutic decision-making in PD patients.

Non-pharmacologic management

Non-pharmacologic strategies represent a fundamental component of hypertension management in patients undergoing peritoneal dialysis, primarily focusing on optimization of volume status, sodium balance, and lifestyle-related factors influencing cardiovascular risk.

Volume Control

Role of achieving dry weight: Achieving and maintaining an appropriate dry weight is a cornerstone strategy for blood pressure control in patients undergoing peritoneal dialysis. It is necessary to maintain optimal volume status in order to treat BP. Measurement by the application of a bioelectrical impedance device is a great way of obtaining dry weight [66]. In a meta-analysis of 42 cohort studies (including data of 60,790 dialysis patients), a BIA-derived >15% overhydration index was correlated with a 2.28-fold increased risk of all-cause mortality [66]. In severely hypertensive patients, it is advisable to decrease the dry weight in steps of 0.5 to 1.0 kg/week. A diet low in sodium, proper management of icodextrin, minimal exposure of the peritoneal membrane to bioincompatible solutions, and adjustment of the PD regimen to the peritoneal transport status are first-line therapeutic interventions for optimal volume control with possible long-term effect on technique survival. Antihypertensive drug therapy is a second-line treatment regimen employed if BP does not respond to the above volume management strategies [67].

Adjustment of dialysate concentration: The use of high-sodium dialysate in treatment prescriptions enhances fluid removal and improves hemodynamic stability. However, it may also lead to increased thirst, greater interdialytic weight gain, and higher fluid removal requirements during subsequent dialysis sessions, thereby creating a potential cycle of worsening fluid balance. Poor BP control could result in some patients [68].

This cycle may be interrupted by tailoring the sodium concentration of the dialysate, which can improve blood pressure control. A nonrandomized trial demonstrated improvement in nocturnal mean arterial pressure among peritoneal dialysis patients receiving low-sodium dialysate. In group A ([Na+] = 115 mmol/L), glucose concentration was adjusted to 2.0% to offset reduced osmolality, whereas in group B ([Na+] = 102 mmol/L), glucose concentration remained unchanged at 2.5%. In both groups, the intervention replaced one daily 3-5-hour exchange, while the remainder of the dialysis regimen remained unchanged.

Both treatment approaches produced substantial increases in diffusive sodium removal during exchange tests (30-50 mmol/dwell; P<0.001). Ultrafiltration was maintained in group A but decreased in group B. Ambulatory nocturnal mean blood pressure decreased in group A (93.1 ± 10.6 mmHg vs 85.1 ± 10.2 mmHg, P < 0.05) but remained unchanged in group B (95.4 ± 9.4 vs 95.1 ± 10.7 mmHg). The combined use of low-sodium dialysate and reduction of dry weight may further improve blood pressure control beyond a single intervention [62].

Role of dwell time: It is well established that peritoneal dialysis (PD) efficiency is dependent on the dwell duration and on the fill volume prescribed. In a randomized crossover trial conducted with varying dwell times, controlled by automated peritoneal dialysis (APD), with conventional methods labelled as APD-C and the implied methods labelled as APD-A, it was observed that the blood pressure was significantly reduced in patients receiving peritoneal dialysis with longer dwell times. Mean BP was not variable at the beginning and end of the APD-C period in the patients initiating with APD-C (group 2: 106.3 ± 7.9 mmHg and 104.9 ± 6.8 mmHg respectively) and mean BP, however, fell significantly from the beginning and end of the APD-A period (106.4 ± 9.6 mmHg and 101.3 ± 8.4 mmHg respectively, p < 0.01, p = 0.0065) [69].

Use of icodextrin solution: Icodextrin dialysate was found to increase ultrafiltration, especially during extended abdominal retention in high peritoneal transport capacity patients. It is a somewhat inert drug with slow absorption, thus providing an osmotic gradient as well as sustained ultrafiltration [70].

A study evaluating the effects of icodextrin on blood pressure in patients undergoing peritoneal dialysis found that twice-daily icodextrin therapy significantly reduced left ventricular mass index (LVMI) by the third month (p < 0.05), with a similar significant reduction also observed in the once-daily icodextrin group (p < 0.05). Twice daily treatment with icodextrin reduced the patients' mean blood pressure significantly (p < 0.05) [71].

Dietary Sodium Restriction

Current recommendations suggest that older individuals and individuals with CKD are most likely to benefit from the reduction of dietary sodium. The reduction of dietary sodium to a maximum of 1.5 g sodium (or about 65 mmol) per day is now recommended [72].

In the literature, there are several studies verifying the positive impact of salt restriction on BP and volume regulation. Excess limitation on fluid intake and salt consumption will reduce the dialysis dose, need to utilize hypertonic solution, and BP [73].

Physical Activity and Lifestyle Modification

The most common cause of death in PD patients, as in CKD and HD patients, remains cardiovascular events. CKD is associated with a risk 18-20 times greater than the risk for cardiovascular disease, still further exacerbated after the start of dialysis therapy. Consistent evidence supports the assertion that an exaggeration of cardiovascular morbidity can be attributed to possibly reversible risk factors like sedentarism, nutritional state, dyslipidemia, and hypertension. Therefore, interventions specifically to change such factors are essential [74].

Pharmacologic management

First-line Agents

ACE inhibitors and ARBs: ACE inhibitors and angiotensin receptor blockers (ARBs) act on the renin-angiotensin-aldosterone system and are widely used for blood pressure control in patients undergoing peritoneal dialysis. By reducing angiotensin II activity, these agents promote vasodilation and decrease sodium and water retention. ACE inhibitors are preferred over other anti-hypertensives, as it has recently been shown that the better the initial protein-lowering response is, the better the renal outcome will be [75]. A study performed to assess the impact of ACE inhibitors showed that during the first eight weeks of treatment with lisinopril, protein excretion was reduced by 61% (± 40%), whereas no such response was observed with conventional therapy [76].

ACE inhibitors have also been associated with regression of left ventricular hypertrophy. Angiotensin II is also known to carry out protein synthesis in the myocytes, so blocking it hinders the process of hypertrophy. ACE inhibitors are also known to block certain sympathetic influences [77].

Due to their role in targeting RAAS, ACE inhibitors pose a risk of hyperkalemia, and this risk intensifies in patients with underlying renal disease. Some important considerations, like intake from diet, drugs, and supplements, can increase serum potassium. If hyperkalemia develops, quick measures should be taken, like recognition of cardiac dysrhythmias to stop the lethal effects of excess potassium on cardiac cells, removal of potassium from the body, and redistribution of potassium into cells. Managing it promptly can improve the clinical outcome [78].

Both ACEis and ARBs are considered first-line agents in peritoneal dialysis patients, but there is not enough supporting evidence available to prove their superiority over other anti-hypertensives regarding residual kidney function. Randomized controlled trials (RCTs) and quasi-RCTs comparing ACE inhibitors or ARBs with each other, placebo, or other conventional anti-hypertensives in peritoneal dialysis patients were included [79].

Calcium channel blockers (CCBs): CCBs offer effective BP reduction without compromising renal perfusion. Animal models suggest that non-dihydropyridine CCBs may provide superior renoprotection by reducing mesangial expansion and glomerular scarring compared to dihydropyridines [80, 81].

Second-line Agents

Beta-blockers: Like RAAS system activation, prolonged compensatory neurohormonal activation has detrimental effects. Blocking the over-activated sympathetic nervous system has been shown to prolong survival in general heart failure cohorts, a benefit that likely translates to the PD population [82, 83]. Beta-blockers are also known to reverse the left ventricular hypertrophy, which plays a part in heart failure. Drugs like bisoprolol, carvedilol, and metoprolol have demonstrated efficacy in reducing all-cause mortality by 34% to 35% in large trials [83]. Extrapolating from broader CKD and heart failure trials, data regarding the effect of beta-blockers on mortality showed a 31% reduction in the odds of death (Odds Ratio [OR] 0.69; 95% CI 0.56-0.86; p<0.01) [83]. Diuretics may help maintain urine output in patients undergoing continuous ambulatory peritoneal dialysis (CAPD), particularly in those with preserved residual kidney function, although this function typically declines over time. Loop diuretics are believed to be beneficial in this regard.

To study this claim, a group of 61 patients at the time of CAPD was randomly assigned either to furosemide 250 mg every day or no furosemide. Diuretics are sometimes used in patients presenting with oliguria in acute renal failure (ARF) to increase the urine output. Studies have shown beneficial effects of loop diuretics, but clinical trials have shown detrimental effects on kidney function [83-86].

Mineralocorticoid receptor antagonists (MRAs): MRAs have been shown to have beneficial effects on myocardial ischemia-reperfusion injury and postinfarction remodeling. Experiments conducted on animal models have shown a significant reduction in the infarct size with MRA administration before ischemia or immediately at the moment of coronary reperfusion. This is believed to be mediated by an intracellular signaling pathway, including adenosine receptor stimulation and activation of several components of the Reperfusion Injury Salvage Kinase (RISK) pathway. MR receptors are expressed by various cells, including the myocytes and endothelial cells. These agents can also promote scar healing if given right after reperfusion and limit the remodeling post-infarction [87, 88]. MRA agents have a downside due to the risk of hyperkalemia associated with them. A cohort study was conducted to assess the risk of subsequent 30-day mortality, and rates of hyperkalemia were compared across strata of estimated GFR and MRA treatment based on Cox regression.

Resistant Hypertension

Clonidine is an older agent with various side effects like dry mouth and sedation, which makes it less favorable as compared to moxonidine, which has fewer side effects due to reduced activity at alpha 2 adrenergic receptors. Instead, it has effects at the cellular site known as I1 imidazoline receptor [89]. Moxonidine has the main effects on lowering blood pressure by affecting the systemic vascular resistance, but poses no effect on heart rate, cardiac output, stroke volume, and pulmonary artery pressure. LVH has been shown to be reduced after six months of treatment [90, 91]. The efficacy of moxonidine as an add-on drug in resistant hypertension was investigated in elderly patients with poor blood pressure control (Table 4).

**Table 4:** Drugs and accompanying study results. ACE: Angiotensin converting enzyme; ARB: Angiotensin receptor blocker; CAPD: Continuous ambulatory peritoneal dialysis; CVMM: Cardiovascular morbidity and mortality; DHP: Dihydropyridine; AHR: Adjusted hazard ratio; ACM: All-cause mortality.

Drugs	Study Results
ACE-Inhibitors and ARBs	ARBs preserve residual kidney function in CAPD with MD 1.11 mL/min/1.73 m^2^, 95% CI -0.75 to -0.11, ACE-Inhibitors show MD -0.93 mL/min/m^2^, 95% CI -0.75 to -0.11, and anuria rate (RR 0.64, 95% CI 0.41 to 0.99) [80].
Calcium channel blockers	AHR of 0.77 (99% CI, 0.65-0.93, P=0.0004) for ACM and 0.86 (0.72-1.02, P=0.024) for CVMM. Results showed DHPs were associated with reduced mortality and morbidity vs non-DHPs [82].
Beta-blockers	Absolute reduction in mean annual mortality from 9.7% to 7.5%. Carvedilol showed 47% reduction, SD 15 while the metoprolol effect on mortality was 18% SD15 [92].
Diuretics	Patients receiving diuretic therapy demonstrated an increase in urine output compared with controls, with changes of +176 mL/24 hours versus −200 mL/24 hours at 6 months and −305 mL/24 hours at 1 year (P < 0.05) [86].
Mineralocorticoid receptor blockers	Among patients with eGFR values of 60, 45–59, 30–44, and <30 mL/min/1.73 m², the hazard ratios (95% CI) were 8.28 (7.78–8.81), 5.12 (4.67–5.62), 3.58 (3.23–3.97), and 1.89 (1.60–2.23), respectively. Mortality rates were lower in patients receiving mineralocorticoid receptor antagonists (MRAs), with absolute risks of 29.3%, 20.3%, and 19.5%, compared with 39.8%, 32.2%, 28.8%, and 22.5% in those not receiving MRA therapy [89].
Moxonidine	Reduction in mean systolic pressure from 163.0 to 148.6 mmHg and mean diastolic pressure from 87.2 to 80.2 mmHg [90].

Special considerations in peritoneal dialysis patients and clinical recommendations

There is always an increased chance of uncontrolled blood pressure in patients undergoing peritoneal dialysis (PD) despite using anti-hypertensive medication. We must consider special circumstances when treating patients with residual renal function and volume overload, as sodium and water retention become a major problem due to inefficient peritoneal transport, especially in those with peritonitis, micro-inflammation, prolonged exposure to glucose, and fibrosis of the peritoneal membrane [92, 93].

A dilemma faced by nephrologists is to optimize blood pressure in PD patients or maintain residual kidney function. Initial steps in volume control include, as a general principle, dietary sodium restriction, prescription of the loop diuretic in patients with residual renal function, adjustment of the peritoneal dialysis regimen according to the patient-specific characteristics based on the transport status of the patient, and the Icodextrin prescription for long dwells [7]. Günal et al. reported a 2 kg loss of body weight within four weeks through strict restriction of sodium and fluid, resulting in controlled blood pressure and improvement in left ventricular hypertrophy (LVH). Additionally, hypertonic PD fluid use led patients to lose 4 kg to achieve normotension [94].

Some trial studies compared the use of icodextrin and 1.36% glucose PD dialysate; extracellular water (ECW) decreased by 2.1 L in the icodextrin-treated group. The use of icodextrin increased the ultrafiltration in both studies [95, 96], but one group also suffered from compromised residual renal function (RRF). Probably due to the underhydration status of patients while using icodextrin [37]. Despite a significant reduction in blood pressure, icodextrin-treated patients required few antihypertensive drugs to control their blood pressure [96]. Thus, a BP-lowering effect was seen in icodextrin-treated patients but not in those who were administered glucose-containing solutions [97].

It is extremely important to institute individualized antihypertensive therapy; this is particularly relevant since blockade of the renin-angiotensin-aldosterone system has been shown to decrease all-cause mortality by an astonishing 62% [79, 98]. A post hoc analysis of the Canada-United States study depicted that for each 250 ml increase in urine volume causes a 36% reduction in all-cause mortality [99]. Loop diuretics increase urine output and fractional urinary excretion of sodium, maintaining the fluid-balance, among PD patients with conserved residual kidney function [100].

In PD patients, anti-hypertensive drugs should be used cautiously, pertaining to their residual renal function and volume overload. A meta-analysis of six randomized trials showed that RAAS (renin-angiotensin-aldosterone system) blockers cause a steady decline of RRF (residual renal function) as compared to other anti-hypertensive drugs [79]. Some trial studies, like Ito et al. [101], Lin et al. [102], proposed that add-on-therapy with mineralocorticoid receptor antagonist improves the LVEF (left ventricular ejection fraction), without causing significant hyperkalemia.

Nocturnal hypertension in PD patients is usually caused by sleep apnea, which is common in volume-overload individuals. In the recumbent position, the fluid volume is redistributed from the legs to the chest and neck areas and may cause upper-airway resistance, which can cause obstructive sleep apnea (OSA) [55]. Hypopneic spells lead to nocturnal hypoxemia during the night, triggering sympathetic arousal and causing an increase in blood pressure during the night. Patients with end-stage renal disease (ESRD) with sleep apnea have a seven-fold higher prevalence of resistant hypertension [103]. The most common complication of peritoneal dialysis is peritonitis, with a prevalence of 25% to 50% [104, 105]. Thus, peritonitis is considered an important cause of failure of treatment and switching to hemodialysis. Low serum albumin levels are an important indicator of poor nutrition as well as inflammation in PD patients [106]. Active acute inflammation in an episode of peritonitis can cause high cardiovascular risk and mortality by triggering local and systemic inflammation. Peritonitis causes changes in peritoneal membrane structure and function by vascular damage, tissue-fibrosis, and the presence of activated leukocytes in the peritoneal cavity, which can affect the rate of peritoneal solute transport, causing failure of ultrafiltration [105].

Since the U-shaped relationship between the level of blood pressure and the mortality rate of patients on peritoneal dialysis does not make fixed targets appropriate, it is recommended that dynamic, patient-specific targets based on parameters such as age, frailty, cardiovascular comorbidity, and residual kidney function be established [26, 107]. This is further supported by regular home and clinical monitoring and the use of remote patient management platforms, which allow real-time adjustments of prescriptions and have been associated with improved hemodynamic profiles as well as with reduced medication needs [108, 109].

Establishment of a multidisciplinary team consisting of nephrologists, nurses, dietitians, social workers, and psychologists has been reported to reduce hospitalization by more than 88% [110], while concurrently improving patient knowledge and promoting self-care activities. It also serves to enhance various nutritional indices as well as decrease levels of inflammation [111, 112]. Furthermore, application of monthly testing at the facility level that measures trends in residual kidney function, dialysate volume, target weight, blood pressure, and adherence to dietary and fluid restrictions ensures a relentless cycle of personalized treatment changes that can quickly respond to the unique needs of each patient [109].

Emerging therapies and future directions

In addition to established treatment strategies, several emerging therapies are currently being investigated for their potential to improve blood pressure control and cardiovascular outcomes in patients undergoing peritoneal dialysis.

Use of SGLT2 Inhibitors

In peritoneal dialysis (PD), an emerging therapy is the use of SGLT2 (sodium-glucose co-transporter) inhibitors, primarily targeting the SGLT2 protein located at the kidney’s proximal tubule, inhibiting glucose reabsorption. While SGLT2 inhibitors are established in the broader CKD and heart failure populations, their application in PD is currently considered preliminary. SGLT2 inhibitors provide a sustained reduction in both systolic blood pressure (SBP) and diastolic blood pressure (DBP). Mazidi et al. outlined a weighted mean difference of -2.46 mmHg (95% CI: -2.86 to -2.06 mmHg) in SBP and -1.46 mmHg (95% CI: -1.82 to -1.09 mmHg) in DBP [113].

Enhancement of endothelial function and mitigation of arterial stiffness are additional mechanisms involved in the reduction in blood pressure [114]. Alhwiesh et al. explored additional benefits of the SGLT2 drug in the induction of weight loss (p=0.0337), ultrafiltration (p=<0.01), and serum uric acid (p=0.0341) reduction [115]. Inhibition of the IL-6 (p=0.010), matrix metalloproteinases (p=0.011), and fibronectin 1 (p=0.055) signalling was demonstrated by SGLT2 inhibitors, suppressing inflammation, extracellular matrix turnover, and fibrosis [116]. Despite these promising signals, large-scale PD-specific randomized controlled trials (RCTs) are required to confirm these preliminary findings.

Common safety concerns include euglycemic ketoacidosis (EKA), hypotension, hypoglycaemia, and infections. PD patients already susceptible to catheter infection now have a greater overall risk profile for infections when treated with SGLT2 inhibitors [114].

Non-dialytic Volume Assessment Tools

Lung ultrasound (US), a non-invasive, simple, and radiation-free technique, provides accuracy in dry weight determination. US is able to provide insights for BP management, heart structure evaluation in relation to the volume overload, and evaluation of hydration status in PD patients [117]. The US provides reliable estimates of the Extravascular Lung Water (EVLW), which correlates with the driving force that promotes fluid extravasation into the lung, correlating with the left ventricular filling pressures. US demonstrates as a dynamic parameter for optimization of fluid management [118]. In RKF patients, ultrafiltration volume and a decrease in the B lines sum are strongly correlated, outlining the interstitial lung congestion during the dialysis sessions [119]. US lacks specificity of the B-lines, a radiologic sign of lung interstitium, and cannot discriminate between lung water excess and the fibrotic thickening commonly seen in pulmonary fibrosis [120]. Nevertheless, bedside US poses to be a sensitive tool to evaluate the dynamic changes in the extravascular lung volume, aiming to optimize the volume status in PD patients.

Digital Health and BP Monitoring Apps

Chen et al. were able to discover the efficacy of web-based home blood pressure monitoring (HBPM) in chronic kidney disease [121]. They developed a digital BP management program comprising regular BP measurements being uploaded automatically, remotely, to a cloud system where physicians and patients could access the measurements. The HBPM provides better reproducibility, more prognostic information, and higher diagnostic accuracy, aiding in guidance on chronic hypertension treatment regimens [122]. The program led to better control of the pre-dialysis BP rates, improved patient knowledge and adherence to the regimens [121]. Technical barriers such as infrastructure costs and connectivity could pose a barrier between patients and physicians [123].

Implementing digital applications could be the future direction not just for BP measurement but also for nutrition assessment, symptom logging, but the lack of enough trials limits exploring their complete potential.

Ongoing Clinical Trials and Evidence Gaps

Emerging therapies are currently exploring new dialysis solutions to enhance the outcomes of PD, though these remain in the preliminary investigative phase. Novel studies are exploring the use of L-carnitine and xylitol, such as the study by Masola et al. [124]. L-carnitine and xylitol contain lower glucose levels that protect the peritoneal viability of peritoneal mesothelial cells and vascular cells [124]. They do not exacerbate the inflammation or angiogenesis, consequently reducing the risk of fibrosis in comparison to the conventional glucose-mediated solutions. Clinical trials have confirmed the safety of these solutions for a four-week administration; however, the long-term efficacy and safety profile for hypertension management is still to be determined and is currently classified as preliminary [125, 126].

The addition of alanyl-glutamine (ALAGln) into the PD fluids has been reported to enhance immune cell functioning within the peritoneal white blood cells, consequently restoring the cellular stress responses. Clinical trials have outlined that they improve peritoneal membrane integrity and reduce systemic inflammation in comparison to neutral pH, low-GDP solutions. The risk of peritonitis was also reported to be lower, and future research in this direction could be crucial to reduce the risk of peritonitis [124-126]. As with other novel solutions, the clinical impact of ALAGln on systemic blood pressure control requires further validation in larger PD cohorts.

These emerging approaches may complement existing pharmacologic and non-pharmacologic strategies by improving fluid assessment, optimizing dialysis prescriptions, and enabling more individualized blood pressure management in PD patients.

## Conclusions

Hypertension management in the peritoneal dialysis (PD) patient is difficult due to high fluid and sodium overload and a reduction in residual renal function. Pharmacological therapy alone is usually ineffective since 1/3 of patients will eventually become refractory. Non-pharmacological approaches, including diet, ultrafiltration, and bioimpedance spectroscopy (BIS), continue to be the mainstay. Given the limitations of such studies, individualized and volume-targeted therapy continues to be important. Advances in ambulatory blood pressure monitoring, novel dialysis solutions, and emerging pharmacologic therapies such as SGLT2 inhibitors may further improve hypertension management in PD patients, although additional prospective studies are required. Effective management of hypertension in peritoneal dialysis requires a multifaceted approach that includes optimization of volume status, appropriate pharmacologic therapy, and careful cardiovascular risk assessment.
